# Elevated α-Ketoglutaric Acid Concentrations and a Lipid-Balanced Signature Are the Key Factors in Long-Term HIV Control

**DOI:** 10.3389/fimmu.2022.822272

**Published:** 2022-04-20

**Authors:** Jenifer Masip, Norma Rallón, Elena Yeregui, Montserrat Olona, Salvador Resino, José M. Benito, Consuelo Viladés, Graciano García-Pardo, José Alcamí, Ezequiel Ruiz-Mateos, Frederic Gómez-Bertomeu, Montserrat Vargas, Marta Navarro, José A. Oteo, Juan A. Pineda, Anna Martí, Verónica Alba, Francesc Vidal, Joaquin Peraire, Anna Rull

**Affiliations:** ^1^ Universitat Rovira i Virgili, Tarragona, Spain; ^2^ Institut Investigació Sanitària Pere Virgili (IISPV), Tarragona, Spain; ^3^ Hospital Universitari de Tarragona Joan XXIII, Tarragona, Spain; ^4^ HIV and Viral Hepatitis Research Laboratory, Instituto de Investigación Sanitaria Fundación Jiménez Díaz, Universidad Autónoma de Madrid (IIS-FJD, UAM), Madrid, Spain; ^5^ Hospital Universitario Rey Juan Carlos, Móstoles, Spain; ^6^ Unidad de Infección Viral e Inmunidad, Centro Nacional de Microbiología, Instituto de Salud Carlos III, Majadahonda, Spain; ^7^ Centro de Investigación Biomédica en Red de Enfermedades Infecciosas (CIBERINFEC), Instituto de Salud Carlos III (ISCIII), Madrid, Spain; ^8^ Instituto de Salud Carlos III, AIDS Immunopathology Unit, National Center of Microbiology, Madrid, Spain; ^9^ HIV Unit, Hospital Clinic-IDIBAPS, Barcelona, Spain; ^10^ Clinical Unit of Infectious Diseases, Microbiology and Preventive Medicine, Institute of Biomedicine of Seville (IBiS), Virgen del Rocío University Hospital, Consejo Superior de Investigaciones Científicas (CSIC), University of Seville, Seville, Spain; ^11^ Servicio de Enfermedades Infecciosas, Parc Tauli Hospital Universitari, Sabadell, Spain; ^12^ Hospital Universitario San Pedro, Centro de Investigación Biomédica de La Rioja (CIBIR), Logroño, Spain; ^13^ Unidad de Investigación Hospital Universitario de Valme, Sevilla, Spain

**Keywords:** metabolomics, lipidomics, elite controllers (ECs), HIV infection, Kreb's cycle, long-term, viral, mass spectrometry

## Abstract

Long-term elite controllers (LTECs) are a fascinating small subset of HIV individuals with viral and immunological HIV control in the long term that have been designated as models of an HIV functional cure. However, data on the LTEC phenotype are still scarce, and hence, the metabolomics and lipidomics signatures in the LTEC-extreme phenotype, LTECs with more than 10 years of viral and immunological HIV control, could be pivotal to finding the keys for functional HIV remission. Metabolomics and lipidomics analyses were performed using high-resolution mass spectrometry (ultra-high-performance liquid chromatography–electrospray ionization–quadrupole time of flight [UHPLC-(ESI) qTOF] in plasma samples of 13 patients defined as LTEC-extreme, a group of 20 LTECs that lost viral and/or immunological control during the follow-up study (LTEC-losing) and 9 EC patients with short-term viral and immunological control (less than 5 years; no-LTEC patients). Long-term viral and immunological HIV-1 control was found to be strongly associated with elevated tricarboxylic acid (TCA) cycle function. Interestingly, of the nine metabolites identified in the TCA cycle, α-ketoglutaric acid (p = 0.004), a metabolite implicated in the activation of the mTOR complex, a modulator of HIV latency and regulator of several biological processes, was found to be a key metabolite in the persistent control. On the other hand, a lipidomics panel combining 45 lipid species showed an optimal percentage of separation and an ability to differentiate LTEC-extreme from LTEC-losing, revealing that an elevated lipidomics plasma profile could be a predictive factor for the reignition of viral replication in LTEC individuals.

## Introduction

HIV infection involves a broad, dynamic process and varies in the different phenotypes of people living with HIV. Elite controllers (ECs) are a fascinating subset of these individuals capable of maintaining viral and immunological control, even during long periods without antiretroviral therapy (ART) (1, 2). This characteristic could make ECs a good pathogenic model for a long-awaited HIV cure (3). However, ECs comprise a heterogeneous population in terms of virological, immunological, and even clinical characteristics. Although previous studies have investigated the mechanisms associated with HIV replication and HIV immunodeficiency that could contribute to the loss of spontaneous control in ECs (transient controllers) (4–6), data on long-term ECs (LTECs) are scarce (7). LTECs are a very small proportion of ECs with viral control (HIV-RNA viral load below 50 copies/ml) and lack immunological progression (positive or null CD4 slope) in the long-term without ART (8). Thus, LTEC subjects have been designated as an appropriate model for long-term HIV remission (3) and pose the controversial query of whether ECs need to receive ART (9).

Based on our previous works studying the loss of spontaneous HIV-1 control in ECs (transient controllers) (4, 5, 10), we firmly believe that the identification and quantification of small molecules, from metabolites to lipids, could provide knowledge of immunotherapeutic strategies for ART-free HIV remission (11) and aid in deciding whether ART is warranted in ECs. Thus, we aimed to define the metabolomics and lipidomics signatures underlying the long-term EC phenotype to understand the mechanisms operating for persistent viral and immunological control in LTEC patients with more than 10 years of HIV control.

## Materials and Methods

### Patient Cohort Enrolment and Study Design

**Figure 1:**
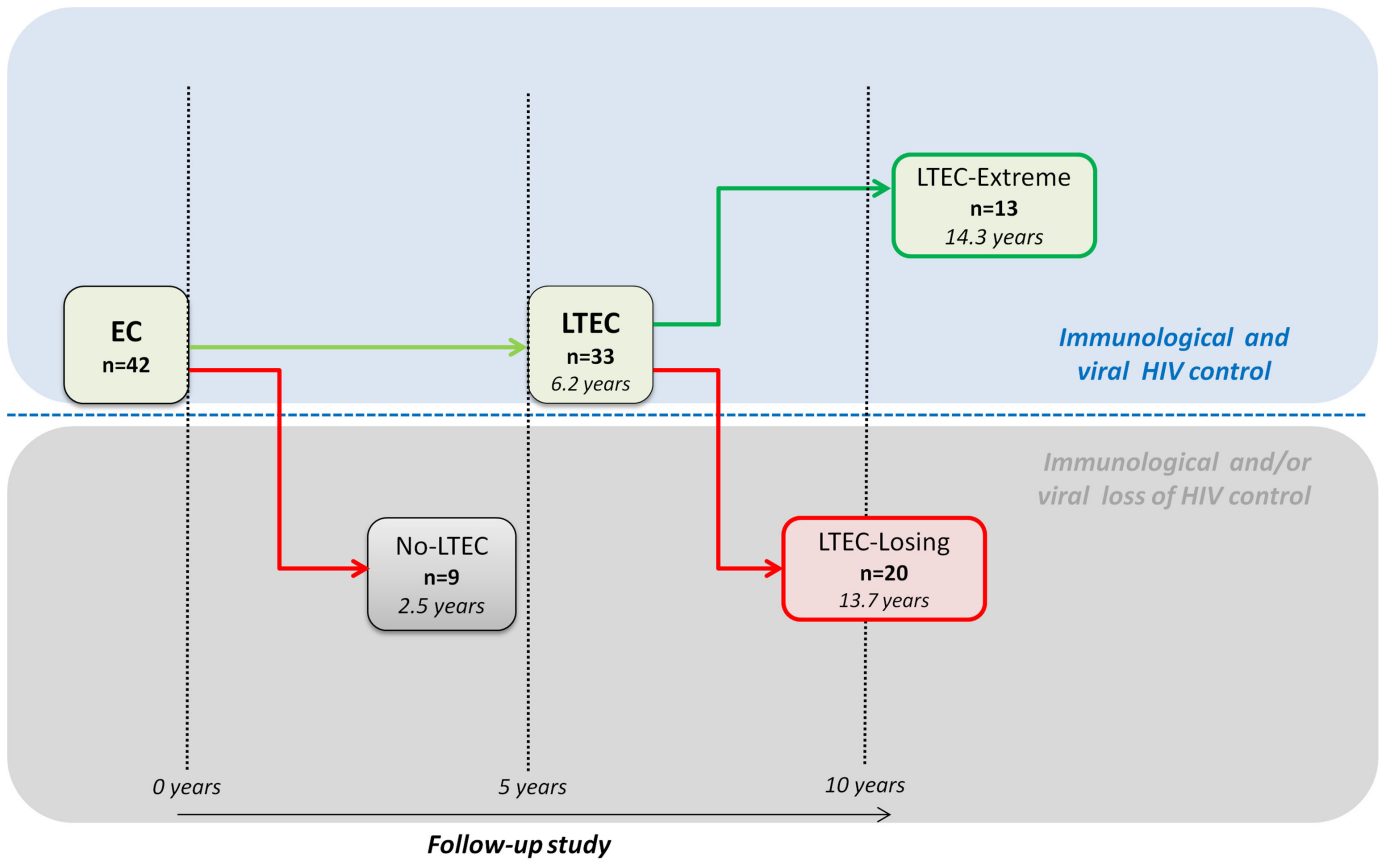
Flowchart illustrating patient cohort enrolment and analysis. From the 42 elite controllers (ECs) analyzed, 9 individuals who experienced a loss of spontaneous viral HIV-1 control in less than 5 years were classified as no long-term elite controllers (no-LTEC); 33 EC individuals who maintained viral and immunological control for at least 5 years were defined as long-term elite controllers (LTECs). Among the LTEC groups, 13 patients were classified as LTEC-extreme (defined as LTECs maintaining viral and immunological control throughout the whole follow-up period and for more than 10 years); and 20 were classified as LTEC-losing (defined as LTECs that lost viral and immunological HIV control during follow-up). Loss of viral control was described as two consecutive measurements of plasma HIV-RNA load above the lower detection limit and a statistically significant negative slope of CD+ T-cell count during the follow-up period.

A total of 42 ECs from the Spanish AIDS Research Network (RIS) cohort of HIV Controllers Study Group (ECRIS) database were retrospectively selected and classified in different EC phenotypes according to their ability to maintain viral and immunological control in the long term (12) (**Figure 1**). Nine EC patients who experienced a loss of spontaneous viral HIV-1 control in less than 5 years of follow-up were classified as no long-term ECs (no-LTEC); 33 EC patients who maintained viral and immunological control for at least 5 years were defined as LTECs. Among the LTEC groups, 13 individuals were classified as LTEC-extreme (defined as LTECs maintaining viral and immunological control throughout the whole follow-up period and for more than 10 years); a group of 20 LTECs that lost viral and immunological HIV control during follow-up were included and compared to the group of LTEC-extreme to find the signature underlying the long-term EC phenotype. Loss of viral control was described as two consecutive measurements of plasma HIV-RNA load above the lower detection limit, and the loss of immunological control was described as a statistically significant negative slope of CD4^+^ T-cell count during the follow-up period (p-value <0.05) (**Table 1**).

**Table 1:** Baseline characteristics of the study participants.

Clinical characteristics	No-LTEC (n = 9)	LTEC-losing (n = 20)	LTEC-extreme (n = 13)	p-Value^*^
**Age (years)**	37 [34–45]	46 [43–50]	48 [42–51]	0.075
**Time of control (years)**	2.5 [1.3–3.3]	13.7 [8.6–17.1]	14.3 [13.2–15.6]	**<0.001**
**Male**	5 (55.6)	8 (40)	9 (69.2)	0.262
**HIV risk factor**				0.141
**Heterosexual**	6 (66.7)	6 (30)	2 (15.4)	
**Homo/bisexual**	1 (11.1)	2 (10)		
**Intravenous drug abuse**	2 (22.2)	10 (50)	10 (76.9)	
**Other/unknown**		2 (10)	1 (7.7)	
**CD4^+^ T-cell count (cells/µl)**	724 [430–1029]	756 [646–1041]	830 [522–1028]	0.772
**CD4 count slope (cells/month)**	–	−0.35 [−1.23 to 0.99]	−1.98 [−2.80 to −1.31]	**0.012**
**HIV viral load (log)**	1.6 [1.45–1.7]	1.7 [1.6–1.7]	1.6 [1.3–1.7]	0.337
**HCV coinfection (positive)**	3 (33.3)	13 (65)	13 (100)	**0.003**
**Active HCV coinfection (positive)**	1 (11.1)	10 (50)	10 (77)	**0.010**

All plasma samples analyzed were obtained before the loss of HIV control (in the case of no-LTEC and LTEC-losing groups) compared to LTEC-extreme. Data are presented as n (%) or median (interquartile range). Categorical data were compared through a χ^2^ test, whereas continuous data were compared using the non-parametric Kruskal–Wallis test^*^. (P values < 0.05 in bold).

no-LTEC, no long-term elite controllers; LTEC-losing, long-term elite controllers patients who lost HIV control during follow-up; LTEC-extreme, long-term elite controllers patients maintaining HIV control during the whole follow-up and for more than 10 years.

### Samples

Samples from patients were kindly provided by the HIV BioBank integrated into the RIS. Briefly, blood samples were collected using ethylenediaminetetraacetic acid (EDTA) tubes and sent on the same day to the Spanish HIV HGM BioBank for processing. Plasma was obtained by centrifugation and stored at −80°C. All plasma samples analyzed were obtained before the loss of HIV control (in the case of no-LTEC and LTEC-losing groups).

### Ethical Protocol

The studies involving human participants were reviewed, approved, and carried out according to the recommendations of the Ethical Committee for Clinical Research following the rules of Good Clinical Practice from the Institut d’Investigació Sanitària Pere Virgili (CEIm IISPV, ref. 041/2018). The CEIM IISPV is an independent committee made up of health and non-health professionals that supervise the correct compliance of the ethical principles governing clinical trials and research projects that are carried out in our region, specifically in terms of methodology, ethics, and laws. All participants in the study gave their written informed consent, and the study protocol was evaluated and approved by institutional Ethical Committees in agreement with the Declaration of Helsinki.

### Determination of the Metabolomics Profile (Analytical Method)

For metabolomics analysis, a protein precipitation extraction was performed by adding eight volumes of methanol:water (8:2) containing internal standard mixture to plasma samples. Samples were mixed and incubated at 4°C for 10 min and centrifuged at 21,000*g*, and the supernatant was evaporated to dryness before compound derivatization [methoxyamine hydrochloride and *N*-methyl-*N*-trimethylsilyltrifluoroacetamide + 1% trimethylsilyl chloride (MSTFA +1% TMCS)]. Samples were analyzed on a 7200 GC-qTOF from Agilent Technologies (Santa Clara, CA, USA). The chromatographic separation was based on the Fiehn method (13), using a J&W Scientific HP5-MS (30 m × 0.25 mm i.d., 0.25 µm) film capillary column and helium as carrier gas using an oven program from 60°C to 325°C. Ionization was done by electronic impact (EI), with electron energy of 70 eV, and operated in full scan mode, recording data in a range between 35 and 700 *m*/*z* at a scan rate of 5 spec/s.

Targeted compounds were identified using pure standards with a mass accuracy of 20 ppm: amino acid mix (Cambridge Isotope Laboratories, Montreal, QC, Canada), pyruvic acid, lactic acid, glycolic acid, 3-hydroxybutyric acid, glycerol, succinic acid, glyceric acid, fumaric acid, malic acid, *d*-threitol, threonic acid, α-ketoglutaric acid, glycerol-1-phosphate, citric acid, *d*-mannitol, myo-inositol, *d*-sucrose, and α-tocopherol (Sigma-Aldrich, St. Louis, MO, USA). Different internal standards were used to correct signal response: labeled amino acid mix standards (Cambridge Isotope Laboratories), succinic-D_4_ acid, myristic-D_27_ acid, glucose-^13^C_6_, and l-methionine-(carboxy-^13^C, methyl-D_3_) (Sigma Aldrich). Chromatographic peaks were deconvoluted using Unknowns Analysis software (version B.09.00, from Agilent, Santa Clara, CA, USA) based on the exact mass. Identification of compounds was tentatively made comparing the mass spectra and retention time of all detected compounds with the Fiehn 2013 Mass Spectral RTL Library and the National Institute of Standards and Technology (NIST) library 11 (2014) libraries also using the Unknowns software. The identity of the main compounds was confirmed with commercial pure standards. After direct (with pure standards) or putative (with library) identification of metabolites, these were semi-quantified in terms of internal standard response ratio. For this relative quantification, the area of specific fragments for each metabolite was divided by the area of its specific internal standard to provide a reliable, accurate, and reproducible relative concentration of metabolites.

### Determination of the Lipidomics Profile (Analytical Method)

Lipidomics is a subset of metabolomics considered a dissimilar discipline due to the uniqueness and functional specificity of lipids relative to other metabolites. A total of 114 lipids were identified in plasma samples. The name of each compound is abbreviated as follows: CE for cholesteryl ester, DG for diacylglycerol, LPC for lysophosphocholine, PC for phosphatidylcholine, SM for sphingomyelin, and TG for triacylglycerol. The first number indicates the acyl carbon atoms, and the second indicates the number of unsaturations.

For the extraction of hydrophobic lipids, liquid–liquid extraction with chloroform:methanol (2:1) based on the Folch procedure was performed by adding four volumes of chloroform:methanol (2:1) containing internal standard mixture (Lipidomic SPLASH^®^ Avanti Polar Lipids, Birmingham, AL, USA) with 15:0-18:1(d7) PC, 18:1(d7) LPC, 18:1(d7), Chol Ester, 15:0-18:1(d7) DG, 15:0-18:1(d7)-15:0 TG and 18:1(d9) SM, among other lipid species) to plasma. Then, the samples were mixed and incubated at −20°C for 30 min. Afterward, 1/10 volumes of NaCl 0.8% were added, and the mixture was centrifuged at 15,000 rpm. The lower phase was recovered, evaporated to dryness, and reconstituted with methanol:methyl-*tert*-butyl ether (9:1) and analyzed on a 1290 Infinity UHPLC coupled to a 6550 qTOF mass spectrometer (Agilent Technologies, Santa Clara, CA, USA) in positive electrospray ionization mode. The chromatographic elution consists of a ternary mobile phase containing water (A), methanol (B), 2-propanol (C), and 200 mM of ammonium formate and 2% formic acid (D). The gradient was as follows: 0 min, 10% B, 35% C, and 5% D; 0.5 min, 10% B, 45% C, and 5% D; 1.5 min, 9.5% B, 47.7% C, and 5% D; 1.6 min, 7.5% B, 58.5% C, and 5% D; 5 min, 7% B, 61.2% C, and 5% D; 5.1 min, 4% B, 77.4% C, and 5% D; 7.5 min, 3.5% B, 80% C, and 5% D; 9 min, 3.5% B, 80% C, and 5% D; 9.5 min, 0% B, 100% C, and 0% D; 11.5 min, 0% B, 100% C, and 0% D; 11.6 min, 10% B, 35% C, and 5% D; and 14 min, 10% B, 35% C, and 5% D. The stationary phase was a C18 column (Kinetex EVO C18 Column, 2.6 μm, 2.1 mm × 100 mm) that allows the sequential elution of the more hydrophobic lipids such as lysophospholipids, SMs, phospholipids, DGs, TGs, and CEs.

To ensure reproducibility during the analysis, a pooled matrix sample was generated by taking a small volume of each experimental sample and was used as a technical replicate throughout the analysis.

The identification of lipid species was performed using the Agilent MassHunter Profinder B.08 software. First, a feature extraction deconvolution was made; then accurate mass and tandem mass spectra, when available, were matched to Metlin-PCDL (2017) from Agilent containing more than 40,000 metabolites and lipids, allowing a mass error of 20 ppm and a score higher than 80 for isotopic distribution. To ensure the tentative characterization, chromatographic behavior of pure standards for each family and corroboration with Lipid Maps database (www.lipidmaps.org) was used to ensure their putative identification. Afterward, matched entities were selected to perform a targeted MS/MS acquisition on the liquid chromatography–quadrupole time of flight–mass spectrometry (LC-qTOF-MS) instrument to corroborate the identification. Lipid species, then, were semiquantified in terms of internal standard response ratio using one internal standard for each lipid family.

### Statistical Analysis

Categorical data were compared through a chi-squared test, whereas continuous data were compared using the Kruskal–Wallis and Mann–Whitney U non-parametric tests. Correlations between variables were assessed using Spearman’s test. Heatmap analysis of hierarchical clustering comparing LTEC phenotypes, by each quantified lipid species concentration, was performed. The Euclidean distance-metric hierarchical cluster represented patients on vertical lines and candidate lipid families on horizontal lines. The scale from blue (low concentration) to red (high concentration) represents the normalized abundance in arbitrary units. The diagnostic accuracy for predicting individuals belonging to different LTEC phenotypes was evaluated by logistic regression and receiver operating characteristic (ROC) curve analysis.

For metabolomics and lipidomics data analysis, log-transformation was applied to all quantified metabolites to normalize the concentration distributions. Quantitative enrichment analysis (QEA) and pathway analysis were performed using the web-based analytical pipeline MetaboAnalyst 5.0 (14) and log-transformed normalized for uni- and multivariate analyses, high-dimensional feature selection, clustering and supervised classification, functional enrichment, and metabolic pathway analysis. Normalized data were then analyzed using the database Kyoto Encyclopedia of Genes and Genomes (KEGG) (15). Significantly altered metabolites were defined by a t-test analysis with a p-value <0.05 and a false discovery rate (FDR) ≤0.05.

The statistical software used was SPSS Software v22 and XLSTAT 2020.5.1.1064. Graphs were generated using GraphPad Prism 5.0. Illustrations were created using the BioRender web server and the web-based analytical MetaboAnalyst 5.0 as indicated in the figure legends. The results were considered statistically significant at p < 0.05.

## Results

### Characteristics of the Study Participants

A total of 42 HIV-EC were recruited based on the study design shown in **Figure 1**. The clinical and demographic characteristics of patients included in the study are summarized in **Table 1**.

No differences were observed in sex, CD4^+^ T-cell counts, or HIV viral load (log) in the LTEC-extreme group compared to the LTEC-losing group or the no-LTEC group. However, LTEC-extreme patients were older (p = 0.036) and showed significantly different transmission routes (p = 0.031) than no-LTEC patients. Additionally, our results revealed a higher prevalence of HCV coinfection in the LTEC-extreme group than in the no-LTEC (p = 0.001) and LTEC-losing (p = 0.018) groups. This higher prevalence of HCV coinfection in the LTEC-extreme group is mainly due to the period of the infection (more than 10 years ago) when the HIV risk factor in this cohort was the majority due to intravenous drug abuse.

### Tricarboxylic Acid Cycle Metabolites Were Increased in the Long-Term Elite Controller-Extreme Group Compared to the No-Long-Term Elite Controller Group

**Figure 2:**
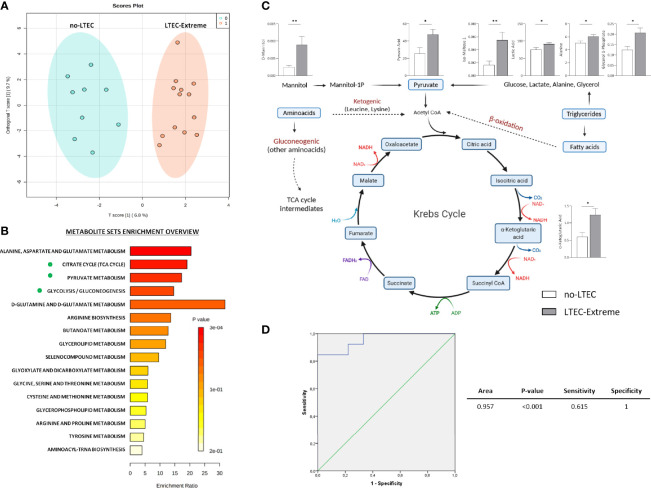
Metabolic analysis comparing long-term elite controller (LTEC)-extreme and no-LTEC. **(A)** Orthogonal principal component analysis (ortho-PCA) of the 78 metabolites between LTEC-extreme and no-LTEC. **(B)** Metabolic enrichment pathways associated with the metabolites differentially expressed in the LTEC-extreme group [Kyoto Encyclopedia of Genes and Genomes (KEGG) database]. The x-axis indicates the impact of selected metabolites in the presented pathway, while the y-axis shows the level of enrichment of the pathway. **(C)** Illustration of the principal significant metabolites between LTEC-extreme and no-LTEC in the tricarboxylic acid (TCA) cycle (column bars indicating differences in relative plasma concentrations of those metabolites implicated in the Krebs cycle; white bar represents no-LTEC; gray bars represent LTEC-extreme [mean + SEM data]. *P < 0.05; **P < 0.01 (adapted from “Kreb’sCycleTemplate,” by BioRender.com (2021); retrieved from https://app.biorender.com/biorender-templates). **(D)** Logistic regression and receiver operator characteristic (ROC) curves elucidated the statistically significant metabolomics profile from the combination of 7 statistically significant TCA cycle metabolites as main differentiators between LTEC-extreme and no-LTEC-losing [area under the curve (AUC) = 0.957].

A total of 78 metabolites and 114 lipid species were identified in the plasma samples of the study cohort (**Supplementary Tables S1**, **S2**). Orthogonal principal component analysis (ortho-PCA) of these 78 metabolites showed a high discriminatory ability of metabolomics profile to separate between the LTEC-extreme and the no-LTEC groups (**Figure 2A**). Indeed, relative plasma concentrations of nine metabolites were significantly higher in the LTEC-extreme group than in the no-LTEC group. Metabolic enrichment pathways were evaluated. The metabolomics profile associated with LTEC-extreme confirmed that the TCA cycle (p = 0.003), pyruvate metabolism (p = 0.004), and glycolysis/gluconeogenesis (p = 0.005) (**Figure 2B**) pathways were among the most enriched pathways related to persistent viral and immunological control. Then, among the significant metabolites in the LTEC-extreme group, we found that seven of them were strongly related to the tricarboxylic acid cycle (TCA cycle) (**Figure 2C**), which could indicate that the Krebs cycle is highly associated with viral control in LTEC-extreme patients. In fact, the combination of these seven metabolites could differentiate perfectly both groups of LTEC phenotype (area under the curve (AUC) = 0.957) (**Figure 2D**).

On the other hand, the ortho-PCA including the 114 lipids identified was unable to differentiate LTEC-extreme from no-LTEC. No significant differences were found between LTEC-extreme and no-LTEC.

### The Important Role of Lipid Species in Continuous Viral and Immunological HIV Control in Long-Term Elite Controllers

**Figure 3:**
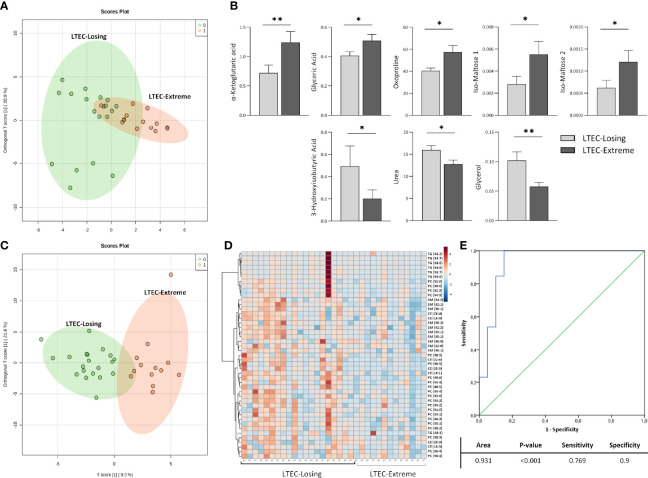
Metabolomics and lipidomics analysis comparing long-term elite controller (LTEC)-extreme and LTEC-losing. **(A)** Orthogonal principal component analysis (ortho-PCA) including 78 metabolites between LTEC-extreme and LTEC-losing. **(B)** Column bars indicating differences in relative plasma concentrations of the principal significant metabolites (*P < 0.05; **P < 0.01) between LTEC-extreme and LTEC-losing. Light gray bars represent LTEC-losing, and dark gray bars represent LTEC-extreme (mean + SEM data). **(C)** Ortho-PCA of the 114 lipid species differentiating the LTEC-extreme from the LTEC-losing group. **(D)** Hierarchical clustering of the 45 significantly expressed lipids species between LTEC-extreme and LTEC-losing. Patients were ordered on vertical lines and candidate relative concentration of each lipid species on horizontal lines [sphingomyelin (SM), phosphatidylcholine (PC), triacylglycerol (TG), and cholesteryl ester (CE)]. The scale shows blue (low concentration) to red (high concentration). **(E)** Logistic regression and receiver operator characteristic (ROC) curves elucidate the statistically significant lipidomics profile from the combination of 45 statistically significant lipid species as main differentiators between LTEC-extreme and LTEC-losing (area under the curve (AUC) = 0.931).

Then, the metabolomics and lipidomics profiles associated with LTEC-extreme were compared to those associated with LTEC-losing. In this case, ortho-PCA including the 78 metabolites identified was unable to offer a clear differentiation among groups, which indicated great metabolomics similarity among the two phenotypes of LTEC (**Figure 3A**). Nevertheless, again, higher relative plasma concentrations of metabolites directly related to the TCA cycle were associated with the LTEC-extreme phenotype. Specifically, plasma levels of α-ketoglutaric acid (p = 0.006), glyceric acid (p = 0.04), oxoproline (p = 0.027), and iso-maltoses 1 and 2 (p = 0.027 and 0.03) were significantly higher in the LTEC-extreme individuals than in the LTEC-losing individuals. Of interest, plasma glycerol (p = 0.006), 3-hydroxyisobutyric acid (3-HIBA) (p = 0.048), and urea (p = 0.036) relative concentrations were significantly higher in the LTEC-losing group than in the LTEC-extreme group (**Figure 3B**).

On the other hand, ortho-PCA including the 114 lipids identified offered good differentiation among the LTEC-extreme and LTEC-losing groups (**Figure 3C**). In agreement with the ortho-PCA, 45 lipid species were statistically significant between the LTEC-extreme and LTEC-losing samples (**Figure 3D**). Lipids from the SM, PC, TG, and CE families were significantly decreased in the LTEC-extreme group compared to the LTEC-losing group. In fact, the lipidomics profile including these 45 lipid species resulted in a good lipidomics panel with an optimal percentage of separation and an ability to differentiate LTEC-extreme from LTEC-losing (AUC = 0.931) (**Figure 3E**).

### Metabolomics/Lipidomics Signatures Related to Long-Term Elite Controller-Extreme Could Be Potential Biomarkers of Long-Term Elite Controller Progression

**Figure 4:**
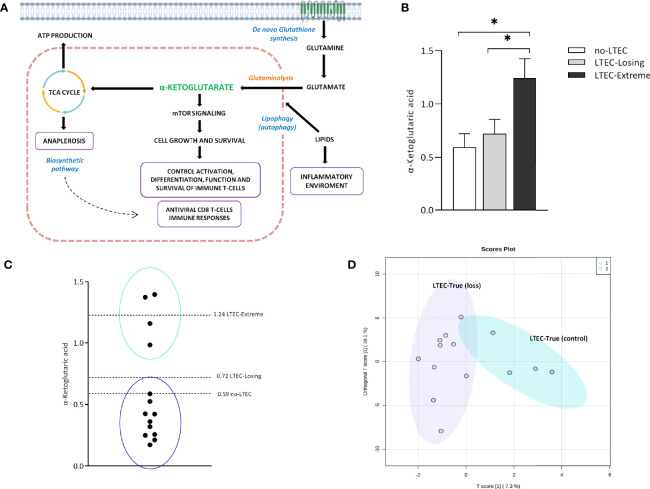
α-Ketoglutaric acid and the lipid panel distinguish two subgroups in long-term elite controller (LTEC)-true. **(A)** Overview of the principal α-ketoglutarate pathways implicated in the antiviral state in the LTEC-extreme patients: mTOR signaling for the control of T immune cells and the biosynthetic pathways in the tricarboxylic acid (TCA) cycle associated with the cell growth and survival. Right: the possible inflammatory environment created by increased lipidic concentrations as the mechanism operating behind the loss of immunological and/or viral control in LTEC-losing patients (created with BioRender.com (2021); retrieved from https://app.biorender.com/biorender-templates). **(B)** Increased plasmatic levels of α-ketoglutaric acid in LTEC-extreme compared to other LTEC phenotypical groups (*P < 0.05). **(C)** Relative plasmatic concentrations of α-ketoglutaric acid distinguish the classification of two subgroups among the 14 LTEC-true individuals. **(D)** Orthogonal principal component analysis (ortho-PCA) of the combination of 45 lipid species significantly increased in the LTEC-losing group differentiates both subgroups of LTEC-true.

Finally, to test that the above-described metabolomics and lipidomics signatures could predict the clinical outcome in LTECs, we used the 14 LTEC individuals, defined as LTEC-true, with viral and immunological control during all the follow-up studies that could not be classified as LTEC-extreme or LTEC-losing, due to missing follow-up until 10 years (6.2 ± 0.38 years). Increased plasmatic concentrations of α-ketoglutaric acid, a product of glutaminolysis (**Figure 4A**), were the most representative of persistent natural HIV control in LTEC-extreme (**Figures 2A**, **3B**). In fact, relative plasma α-ketoglutaric acid concentrations were significantly higher in LTEC-extreme individuals than in both LTEC-losing and non-LTEC individuals (**Figure 4B**). Hence, we used plasma α-ketoglutaric acid concentrations as a potential distinctive metabolite of the 14 LTEC-true individuals. Our results revealed 4 LTEC-true subjects showing relative plasma α-ketoglutaric acid concentrations around the plasma mean α-ketoglutaric acid relative concentrations previously described for LTEC-extreme (1.24 ± 0.66) (**Figure 4C**). Accordingly, we classified two subgroups of individuals, a group of 4 LTEC-true with the potential capability to become LTEC-extreme and a group of 10 LTEC-true who could probably become LTEC-losing (**Figure 4C**). Thus, these two new groups of LTEC-true were evaluated using an ortho-PCA, which included the lipidomics panel of 45 lipid species related to the spontaneous loss of viral and/or immunological HIV control in LTEC-losing (**Figures 3D** **, E**). As illustrated in the PCA representation (**Figure 4D**), the LTEC-true group with the potential capability to maintain viral and immunological HIV control and the LTEC-true group that could probably become LTEC-losing showed clear differentiation. Altogether, these data indicate the feasibility of plasma α-ketoglutaric acid concentrations and the panel of 45 lipid species, including the SM, PC, TG, and CE families, as potential biomarkers for the loss of viral and/or immunological control in LTEC individuals.

## Discussion

LTECs are a heterogeneous group of ECs representing subjects who maintain viral control with a stable CD4^+^ immunological state for more than 10 years (1, 16) as well as patients who occasionally lose HIV control at some point in their progression. Multiple studies have already confirmed that metabolic pathways can regulate innate and adaptive host responses to infections (17), but substantial heterogeneity exists in ECs in terms of virological, immunological, and clinical outcomes. Thus, data on EC phenotypes, such as LTECs, have not yet been fully described. Hence, in this work, our objective was to elucidate the metabolic and lipidomics profiles related to LTEC-extreme to fully understand the mechanism implicated in persistent viral and immunological HIV control. To our knowledge, this extraordinary LTEC phenotype would be useful in the search for a model of functional cure and to decide whether ART is warranted in ECs.

Previously, an immunometabolism study of EC described the strong associations among metabolites, lipid levels, and important immune function parameters associated with spontaneous control of HIV (4). However, the current study identified for the first time metabolomics and lipidomics signatures associated with the persistent viral and immunological control of HIV-1 infection. Some important metabolic pathways described in this article related to continuous viral and immunological HIV control are glycolysis, the TCA cycle, fatty acid metabolism (FAO), and amino acid metabolism (glutaminolysis) (18).

Metabolic pathways related to disturbances in energy metabolism, such as glycolysis, the TCA cycle, and amino acid catabolism, have been previously related to viral control in ECs (19). Previous studies have demonstrated that all of these pathways are targeted during viral infection, characterized by a high demand for energy (20), a decrease in circulating metabolite levels such as glucose, and an increased profile of glycolytic intermediates (glycerol 3P, pyruvate, etc.) (21) in HIV-infected CD4^+^ T cells. Accordingly, our results demonstrated that there was a specific metabolomics profile associated with persistent immunological and viral control in the LTEC-extreme phenotype that was strongly related to critical deregulation of the TCA cycle.

Of note, relative plasma concentrations of α-ketoglutaric acid, a key molecule in the Krebs cycle determining the overall rate of the citric acid cycle in the organism, were significantly increased in LTEC-extreme compared to no-LTEC and LTEC-losing (**Figure 4B**). Indeed, plasma concentrations of α-ketoglutaric acid showed good clustering within LTEC-true individuals, which could predict the natural evolution of persistent HIV control. In glutamine metabolism, α-ketoglutarate activates mTOR, a catalytic factor of key cellular pathways regulating cell growth and metabolism that control T-cell activation, differentiation, function, and survival (22). In fact, mTOR has been described as a modulator of HIV latency in Th17 cells in the use of mTOR inhibitors as a potential therapeutic option in decreasing HIV reservoirs and restoring the Th17-mediated immunity at the intestinal level during ART (23, 24). Moreover, increased levels of α-ketoglutaric acid could indicate an antiviral maintained state that impedes HIV replication since the incorporation of α-ketoglutaric acid into the TCA cycle is the major anaplerotic step in proliferating cells (25) (**Figure 4A**). Furthermore, some studies have proposed that cytotoxic T cells have an important and relevant role in immunological viral control in ECs (specific CD8^+^ T-cell transcriptional profiles in ECs) (26). Although studies failed to sustain a specific antiviral effect of CD8^+^ T cells in ECs, they described activation of related metabolic pathways governed by PI3K/AKT, mTOR, and eIF2, which exhibited regulation of cellular growth, proliferation, and metabolism, as previously mentioned. In this sense, Loucif et al. found an association between lipophagy (degradation of endogenous lipids *via* autophagy) as a critical immune mediator to induce functional antiviral CD8^+^ T-cell responses that have an important role in the natural control of HIV-1 infection in ECs (27). Concretely, enhanced lipophagy leads to fuel mitochondrial metabolism due to glutaminolysis and restores protective CD8A T-cell immunity during persistent HIV-1 infection in an IL-21-dependent manner (28, 29). Thus, the plasmatic accumulation of TCA metabolites in LTEC-extreme not only suggests that in proliferating cells the TCA cycle operates as a biosynthetic pathway (anaplerosis) (30, 31) instead of as a purely bioenergetic pathway (**Figure 2B**), but it could also reflect proliferative and survival states of CD8^+^ T cells as a result of different immune mechanisms underlying natural HIV-1 control. Paradoxically, α-ketoglutarate was one of the increased metabolites in transient controllers before the loss of HIV control compared to the persistent controllers (4). This suggests that α-ketoglutarate could enter the TCA cycle for energy production to compensate for the lack of oxidative Krebs cycle activity. However, the follow-up period of the transient controllers was only 1 year, compared to the LTEC-extreme phenotype, which can maintain viral and immunological control for more than 10 years. On the other hand, although no significant differences were found in the plasma concentration of lipid species when LTEC-extreme was compared to the no-LTEC group, the lipidomics signature (relative concentration of 45 lipid species) was significantly different in LTEC-losing compared to LTEC-extreme. Our results showed a decreased lipidomics profile in the LTEC-extreme phenotype that could provide a high association between increased relative concentrations of lipidic species and the spontaneous loss of viral and/or immunological control (AUC = 0.931, from a panel of 45 lipid species that differentiated LTEC-losing from LTEC-extreme) (**Figure 3D**). Lipid processing and transport are affected by inflammatory processes, and many lipid species contribute to inflammation and immune activation and are essential during T-cell differentiation and immune CD8^+^ T-cell responses (32). However, the relationship between the lipidomics profile and the inflammatory state is complex (33). Among the increased relative plasma concentrations of lipid species that were in the LTEC-losing group, we found TG, PC, and CE. Of note, previous studies suggest an increase in TG concentrations during HIV infection (34, 35), whereas PC indirectly activates the TNF-a signaling cascade (36) *via* PC-derived 1,2-DAG (PC-specific phospholipase C) and acts as a proinflammatory contributor to HIV persistence and rapid post-ART HIV rebound (37). In this sense, El-far et al. previously described the important role of the proinflammatory cytokine IL-32 as a powerful biomarker for control failure in HIV-infected slow progressor subjects. Circulating levels of IL-32 positively correlated with the decline of CD4 T-cell counts, increased viral load, lower CD4/CD8 ratio, and levels of other inflammatory markers (sCD14 and IL-6) (**Figure 4A**) (38). Another study associated the coinfection with cytomegalovirus (CMV) to additional inflammation, leading to CD4 T-cell activation, which contributes to progressive T-cell loss (CD4 T-cell decay) in ECs (39). Thus, according to our results, an increase in the relative concentration of the lipidic profile in the LTEC-losing group could be associated with a sustained inflammatory environment, which in turn would be very advantageous for HIV replication (**Figure 4A**). Furthermore, the combination of these 45 lipid species was a main differentiating factor in the evaluation of LTEC progression.

Our study has several limitations. Although the number of patients per group did not seem to be consistent, LTEC patients are not common, and it is difficult to have a continuous follow-up of more than 10 years for this EC phenotype. Accordingly, with this long period of follow-up for more than 10 years, most of the LTEC-extreme patients had an active HCV coinfection that, although being treated in most cases, was not cured. In this regard, information regarding HLA typing was missing. Other inflammatory-related parameters (such as CMV status, IL-32, or IL-21), immunological recovery factors (CD4/CD8 ratio), or quantitative concentration of some target mitochondrial fuels would have been very useful in the understanding of mechanistic pathways. On the other hand, the association between metabolomics and lipidomics in previous omics studies in ECs was also challenging because of the high diversity of patients included in the LTEC cohort. Designating relative plasma α-ketoglutaric acid concentrations and the panel of 45 lipid species as potential biomarkers of extreme long-term conditions requires a consistent group of LTEC individuals with continuous follow-up. Validation studies are needed.

To conclude, our study reveals a singular metabolomics profile associated with maintained viral and/or immunological control in LTEC-extreme individuals. Notably, elevated plasma concentrations of TCA metabolites in LTEC-extreme were associated with the natural control of HIV infection, especially those metabolites related to glutamine metabolism. On the other hand, the lipidomics pattern is highly associated with the spontaneous loss of viral and/or immunological control in LTEC individuals, with good differentiation of LTEC-losing versus LTEC-extreme. Although the exact mechanism underlying natural HIV control in LTEC-extreme is not fully described, we can hypothesize the importance of metabolites and/or lipid species in immune metabolism as potential biomarkers among phenotypical groups of LTECs.

## Data Availability Statement

The raw data supporting the conclusions of this article will be made available by the authors, without undue reservation.

## Ethics Statement

All research protocols were approved and carried out according to the recommendations of the Ethical Committee for Clinical Research following the rules of Good Clinical Practice from the Institut d’Investigació Sanitària Pere Virgili (CEIm IISPV, ref. 041/2018). The patients/participants provided their written informed consent to participate in this study.

## Author Contributions

All authors have seen and approved the submitted version of the manuscript. The author’s contributions are as follows: experimental design (JM, NR, SR, GG-P, JB, ER-M, and AR) and intellectual guidance (NR, JB, CV, JA, FG-B, ER-M, MN, JO, JAP, FV, JP, and AR); recruitment of participants (JM, NR, EY, MO, JB, ER-M, MV, AM, FV, and AR) and sample procurement (JM and VA); data collection (NR, JB, ER-M, FV, JP, and AR); data analysis and interpretation (JM, NR, JB, ER-M, and AR); manuscript preparation (JM, NR, JB, ER-M, FV, AR, and JP). JM, NR, JB, ER-M, FV, AR, and JP were responsible for the study design, data analysis, and article development. NR, JB, ER-M, FV, and AR reviewed and edited the manuscript.

## Funding

This work was supported by the Fondo de Investigación Sanitaria [PI13/0796, PI16/00503, PI16/0684, PI18/1532, PI19/00004, PI19/01127, PI19/01337 PI16/001769, PI19/00973, and PI20/00326]-ISCIII-FEDER (co-funded by the European Regional Development Fund/European Social Fund; “A way to make Europe”/”Investing in your future”); Programa de Suport als Grups de Recerca AGAUR (2017SGR948); Gilead Fellowship Program GLD14/293; The SPANISH AIDS Research Network [RD16/0002/0001, RD16/0002/0002, RD16/0025/0006, RD16/0025/0013, and RD16/0025/0020]-ISCIII-FEDER (Spain); and the Centro de Investigación Biomédica en Red de Enfermedades Infecciosas-ISCIII [CB21/13/00015, CB21/13/00020, and CB21/13/00044], Madrid, Spain. JM is supported by the Universitat Rovira I Virgili under grant agreement “2019PMF-PIPF-18,” through the call “Martí Franquès Research Fellowship Programme.” NR is a Miguel Servet researcher from the ISCIII [CPII19/00025]. EY is supported by the Instituto de Salud Carlos III (ISCIII) under grant agreement “FI20/0011800” through the program “Contratos Predoctorales de Formación en Investigación en Salud.” ER-M was supported by the Spanish National Research Council (CSIC). FV is supported by grants from the Programa de Intensificación de Investigadores (INT20/00031)-ISCIII and by “Premi a la Trajectòria Investigadora als Hospitals de l’ICS 2018.” AR is supported by IISPV through the project “2019/IISPV/05” (Boosting Young Talent), by GeSIDA through the “III Premio para Jóvenes Investigadores 2019,” and by the Instituto de Salud Carlos III (ISCIII) under grant agreement “CP19/00146” through the Miguel Servet Program.

## Conflict of Interest

The authors declare that the research was conducted in the absence of any commercial or financial relationships that could be construed as a potential conflict of interest.

## Publisher’s Note

All claims expressed in this article are solely those of the authors and do not necessarily represent those of their affiliated organizations, or those of the publisher, the editors and the reviewers. Any product that may be evaluated in this article, or claim that may be made by its manufacturer, is not guaranteed or endorsed by the publisher.
