# Supplementary material for: Elevated α-Ketoglutaric Acid Concentrations and a Lipid-Balanced Signature Are the Key Factors in Long-Term HIV Control
Source: Front Immunol. 2022 Apr 20;13:822272. doi: 10.3389/fimmu.2022.822272 (PMC9065415; doi:10.3389/fimmu.2022.822272)
Supplement: Supplementary file 1 [file DataSheet_1.pdf]

Supplementary Material

Elevated α-ketoglutaric acid concentrations and a lipid-balanced signature are the key factors in long-term HIV control

Jenifer Masip[1, 2, 3], Norma Rallón[4, 5], Elena Yeregui[2,3], Montserrat Olona[1,2,3], Salvador Resino[6,7], José M Benito[4,5], Consuelo Viladés[1,2,3,7], Graciano García-Pardo[1,2,3], José Alcamí[7,8,9], Ezequiel Ruiz-Mateos[10], Frederic Gómez-Bertomeu[1,3], Montserrat Vargas[1,3], Marta Navarro[11], José A. Oteo[12], Juan A. Pineda[13], Anna Martí[1,2,3], Verónica Alba[1,2,3], Francesc Vidal[1,2,3,7], Joaquin Peraire[1,2,3,7*†], Anna Rull[1,2,3,7*†], On behalf of ECRIS integrated in the Spanish AIDS Research Network

1 Additional information

Table S1. List of the 78 metabolites identified in the study, including Human Metabolome Database ID, Retention time (RT), quantitative and qualitative ions and internal standard used.Table S2. List of the 114 lipid species identified in the study.

Table S2. List of the 114 lipid species identified in the study, including parental ion, *m/z* and retention time.

Table S3. Relative concentrations of the 45 lipid species increased in the LTEC-losing group when compared to the LTEC-extreme individuals.

Supplementary Figure 1. Overlapped extracted ion chromatograms (EIC) of metabolites analyzed by GC-EI-QTOF-MS.

Supplementary Figure 2. Overlapped extracted ion chromatograms (EIC) of lipids analyzed by LC-ESI-QTOF-MS using the accurate mass (m/z) from pseudomolecular ions. LCP, lysophosphatidylcholines; PC, phosphatidylcholines; SM, sphingomyelins; DG, diglyceride; ChoE/CE, cholesteroyl esthers; TG, triglycerides.

2 ECRIS integrated in the Spanish AIDS Research Network

1 Additional information

Table S1. List of the 78 metabolites identified in the study, including Human Metabolome Database ID, Retention time (RT), quantitative and qualitative ions and internal standard used.

Metabolite/Internal Standard	Human Metabolome Database	RT (min)	M^+ (*m/z*) (Quantitative)	M^+ (*m/z*) (Qualitative)	Internal standard
**2-Hydroxybutyric acid**	HMDB0000008	6.87	131.0895	205.106	Succinic-d_4 acid
**2-Hydroxyisobutyric acid**	HMDB0000729	5.91	131.0895	205.106	Succinic-d_4 acid
**2-Hydroxyisovaleric acid**	HMDB0000407	7.39	145.1061	147.0672	Succinic-d_4 acid
**3-hydroxybutyric acid / 3-hydroxyisobutyric acid**	HMDB0000357/ HMDB0062640	7.31	117.0731	191.0925	Succinic-d_4 acid
**3-Hydroxyisovaleric acid**	HMDB0000407	8.02	131.0859	205.1086	Succinic-d_4 acid
**4-Hydroxybenzoic acid**	HMDB0000500	13.41	223.101	267.0912	Succinic-d_4 acid
**4-hydroxyphenyllactic acid**	HMDB0000755	16.26	179.0902	308.116	Succinic-d_4 acid
**4-Hydroxyproline**	HMDB0000725	12.28	230.1434	140.0919	L-Proline $^{13}C_5$
**Alanine**	HMDB0000161	6.45	116.0916	190.1094	L-Alanine $^{13}C_3$
**alpha-Ketoglutaric acid**	HMDB0000208	12.82	198.0622	288.1119	Succinic-d_4 acid
**alpha-Tocopherol**	HMDB0001893	26.30	237.1337	502.4279	Glucose-$^{13}C_6$
**Aspartic acid**	HMDB0000191	12.23	232.13	218.1075	L-Aspartic acid $^{13}C_4$
**Benzoic acid**	HMDB0001870	8.51	179.0559	135.0652	Succinic-d_4 acid
**Citric acid**	HMDB0000094	15.71	273.105	347.1215	Succinic-d_4 acid
**Creatinine**	HMDB0000562	12.61	115.0841	329.1847	L-Proline $^{13}C_5$
**d-Arabinose**	HMDB0029942	14.06	307.1622	277.1497	Glucose-$^{13}C_6$
**Decanoic acid**	HMDB0000511	11.29	229.1615		Myristic-d_{27} acid
**d-Fructose**	HMDB0000660	16.36	307.165	103.06	Glucose-$^{13}C_6$
**d-Galactitol**	HMDB0000107	17.19	319.1675	307.1653	Glucose-$^{13}C_6$
**d-Gluconic acid**	HMDB0000625	17.35	292.1385	333.1434	Glucose-$^{13}C_6$
**d-Maltose**	HMDB0000163	23.72	361.179	204.1134	Glucose-$^{13}C_6$
**d-Maltose (isomer)**	HMDB0000163	23.81	361.179	204.1134	Glucose-$^{13}C_6$
**d-Mannitol**	HMDB0000765	16.95	319.1675	307.1653	Glucose-$^{13}C_6$
**d-Mannonic acid**	HMDB0242119	17.10	333.1434	292.1385	Glucose-$^{13}C_6$
**Dodecanoic acid**	HMDB0000638	13.64	257.1941		Myristic-d_{27} acid
**d-Sucrose**	HMDB0000258	23.18	361.179	204.1134	Glucose-$^{13}C_6$
**d-Threitol**	HMDB0004136	12.16	204.107	117.0412	Glucose-$^{13}C_6$
**d-Xylose**	HMDB0000098	13.89	307.1622	277.1497	Glucose-$^{13}C_6$
**Erythronic acid**	HMDB0000613	12.61	217.1142	117.0401	Glucose-$^{13}C_6$
**Ethanolamine**	HMDB0000149	8.85	174.1147	262.1472	L-Valine $^{13}C_5$
**Fumaric acid**	HMDB0000134	9.93	245.0752	217.0746	Succinic-d_4 acid
**Galactonic acid**	HMDB0000565	17.43	292.1395		Glucose-$^{13}C_6$
**Galacturonic acid**	HMDB0003363	17.18	333.1434	292.1385	Glucose-$^{13}C_6$

**Glutamic acid**	HMDB0000148	13.41	246.1392	230.1061	L-Glutamic $^{13}C_5$
**Glutamine**	HMDB0000641	15.17	156.0847	245.1485	L-Glutamic $^{13}C_5$
**Glyceric acid**	HMDB0000139	9.79	189.0782	292.1363	Succinic-d_4 acid
**Glycerol**	HMDB0000131	9.00	218.1185		Glucose-$^{13}C_6$
**Glycerol-1-phosphate**	HMDB0000126	15.11	299.0801	357.1222	Glucose-$^{13}C_6$
**Glycine**	HMDB0000123	9.41	174.1153	248.1331	Glycine $^{13}C_2$
**Glycolic acid**	HMDB0000115	6.04	177.0764	131.0353	Succinic-d_4 acid
**Heptanoic acid**	HMDB0000666	7.34	187.1148		Myristic-d_{27} acid
**Hexanoic acid**	HMDB0000535	5.934	173.1005		Myristic-d_{27} acid
**Hippuric acid**	HMDB0000714	15.73	105.0373	206.1045	Succinic-d_4 acid
**Indole-3-propanoic acid**	HMDB0002302	18.19	202.1095	333.1607	Succinic-d_4 acid
**Indolelactic acid**	HMDB0000671	19.14	202.1064		Succinic-d_4 acid
**Isoleucine**	HMDB0000172	9.24	158.1404	218.1063	L-Isoleucine $^{13}C_6$
**Lactic acid**	HMDB0144295	5.83	190.088	117.0767	Succinic-d_4 acid
**Leucine**	HMDB0000687	8.94	158.1432	218.1053	L-Leucine 13C6
**Linoleic acid**	HMDB0000673	19.258	117.0382	337.2654	Myristic-d_{27} acid
**Malic acid**	HMDB0000744	11.85	233.1045	245.0686	Succinic-d_4 acid
**meso-Erythritol**	HMDB0002994	12.06	204.107	117.0412	Glucose-$^{13}C_6$
**Methionine**	HMDB0033951	12.15	128.0909	176.0949	Methionine $^{13}C_5$
**myo-Inositol**	HMDB0000211	18.48	318.1569	305.1495	Glucose-$^{13}C_6$
**Nonanoic acid**	HMDB0000847	10.04	215.1464		Myristic-d_{27} acid
**Octanoic acid**	HMDB0000482	8.72	201.1311		Myristic-d_{27} acid
**Oleic acid**	HMDB0000207	19.32	117.0382	339.2795	Myristic-d_{27} acid
**Oleic acid (isomer)**	HMDB0000207	19.3566	117.0382		Myristic-d_{27} acid
**Ornithine**	HMDB0000214	15.63	142.1063	174.1163	L-Glutamic $^{13}C_5$
**Oxalic acid**	HMDB0002329	6.912	131.0353	175.0616	Succinic-d_4 acid
**Oxoproline**	HMDB0000267	12.21	156.0853	230.1032	L-Proline $^{13}C_5$
**Palmitic acid**	HMDB0000220	17.73	117.0382	313.2578	Myristic-d_{27} acid
**Phenylalanine**	HMDB0000159	13.47	218.105	192.1222	L-Phenylalanine $^{13}C_9$
**Phosphoric acid**	HMDB0001488	9.13	299.0788		Succinic-d_4 acid
**Pipecolic acid**	HMDB0000070	10.17	156.122	230.1397	L-Serine $^{13}C_3$
**Proline**	HMDB0000162	9.27	142.1082	216.1261	L-Proline $^{13}C_5$
**Pyruvic acid**	HMDB0000243	5.68	174.0629	89.0438	Succinic-d_4 acid
**Ribonic acid**	HMDB0000867	15.31	292.1424	333.1432	Glucose-$^{13}C_6$
**Saccharic acid**	HMDB0000663	17.68	292.1385	333.1434	Glucose-$^{13}C_6$
**Sarcosine**	HMDB0000271	6.95	116.0909	190.1089	L-Alanine $^{13}C_3$
**Serine**	HMDB0000187	10.18	204.1316	218.1084	L-Serine $^{13}C_3$
**Stearic acid**	HMDB0000827	19.52	117.0382	341.2983	Myristic-d_{27} acid
**Succinic acid**	HMDB0000254	9.46	247.0851	147.0702	Succinic-d_4 acid

**Tetradecanoic acid**	HMDB0000806	15.776	285.2254		Myristic-d_{27} acid
**Threonic acid**	HMDB0000943	12.55	292.1383	220.098	Succinic-d_4 acid
**Threonine**	HMDB0000167	10.55	218.1053	117.0738	L-Threonine $^{13}C_4$
**Urea**	HMDB0000294	8.45	189.0909	171.0786	Glycine $^{13}C_2$
**Uric acid**	HMDB0000289	18.51	441.1685	456.1901	Succinic-d_4 acid
**Valine**	HMDB0000883	8.13	144.1229	218.1131	L-Valine $^{13}C_5$
**L-Alanine $^{13}C_3$**	-	6.40	119.096		-
**L-Valine $^{13}C_5$**	-	8.06	149.1347		-
**L-Leucine $^{13}C_6$**	-	8.86	164.1546		-
**L-Isoleucine $^{13}C_6$**	-	9.17	164.1573		-
**L-Proline $^{13}C_5$**	-	9.16	221.1068		-
**Glycine $^{13}C_2$**	-	9.34	176.1242		-
**L-serine $^{13}C_3$**	-	10.11	207.1323		-
**L-Threonine $^{13}C_4$**	-	10.48	222.1371		-
**L-Methionine-(carboxy-^{13}C, methyl-D_3**	-	12.10	132.0988		-
**L-Aspartic acid $^{13}C_4$**	-	12.16	236.1305		-
**L-Glutamic $^{13}C_5$**	-	13.34	251.1492		-
**L-Phenylalanine $^{13}C_9$**	-	13.40	201.1486		-
**Succinic-d_4 acid**	-	9.34	251.1121		-
**Glucose-$^{13}C_6$**	-	16.64	323.1732		-
**L-Alanine $^{13}C_3$**	-	6.40	119.096		-

Table S2. List of the 114 lipid species identified in the study, including parental ion, *m/z* and retention time.

Lipid	Parent ion	m/z	Retention time (min)
**CE 16:0**	[M+Na]+	647.5738	9.04
**CE 16:1**	[M+Na]+	645.5581	8.62
**CE 17:1**	[M+Na]+	659.5738	8.90
**CE 18:0**	[M+Na]+	675.6051	9.60
**CE 18:1**	[M+Na]+	673.5894	9.10
**CE 18:2**	[M+Na]+	671.5738	8.80
**CE 18:3**	[M+Na]+	669.5581	8.50
**CE 20:2**	[M+Na]+	699.6051	9.30
**CE 20:3**	[M+Na]+	697.5894	8.96
**CE 20:4**	[M+Na]+	695.5738	8.65
**CE 20:5**	[M+Na]+	693.5581	8.40
**CE 22:4**	[M+Na]+	723.6051	9.08
**CE 22:5**	[M+Na]+	721.5894	8.87
**CE 22:6**	[M+Na]+	719.5738	8.59
**DG 34:1**	[M+Na]+	617.5115	6.51
**DG 34:2**	[M+Na]+	615.4959	6.26
**DG 34:3**	[M+Na]+	613.4802	6.14
**DG 36:1**	[M+Na]+	645.5434	6.70
**DG 36:2**	[M+Na]+	643.5272	6.51
**DG 36:3**	[M+Na]+	641.5115	6.34
**DG 36:4**	[M+Na]+	639.4959	6.15
**DG 40:4**	[M+Na]+	695.5591	8.26
**LPC 15:0**	[M+H]+	482.3241	1.47
**LPC 16:0**	[M+H]+	496.3398	1.68
**LPC 16:0 e**	[M+H]+	482.3605	1.86
**LPC 16:1 e**	[M+H]+	480.3449	1.86
**LPC 18:0**	[M+H]+	524.3711	2.13
**LPC 18:0 e**	[M+H]+	510.3918	2.35
**LPC 18:1**	[M+H]+	522.3554	1.79
**LPC 18:2**	[M+H]+	520.3398	1.56
**LPC 20:0**	[M+H]+	552.4024	2.64
**LPC 20:2**	[M+H]+	548.3711	1.94
**LPC 20:3**	[M+H]+	546.3554	1.72
**PC 30:0**	[M+H]+	706.5381	3.80
**PC 31:0**	[M+H]+	720.5538	4.05
**PC 32:0**	[M+H]+	734.5694	4.32
**PC 32:1**	[M+H]+	732.5538	3.96
**PC 32:2**	[M+H]+	730.5381	3.76
**PC 33:0**	[M+H]+	748.5851	4.51

**PC 33:1**	[M+H]+	746.5694	4.21
**PC 33:2**	[M+H]+	730.5381	3.67
**PC 34:0**	[M+H]+	762.6008	4.99
**PC 34:1**	[M+H]+	760.5851	4.50
**PC 34:2**	[M+H]+	758.5694	4.16
**PC 34:3**	[M+H]+	756.5538	3.81
**PC 34:4**	[M+H]+	754.5381	3.71
**PC 35:1**	[M+H]+	774.6007	4.83
**PC 35:2**	[M+H]+	772.5851	4.45
**PC 35:4**	[M+H]+	768.5538	4.21
**PC 36:1**	[M+H]+	788.6164	5.23
**PC 36:2**	[M+H]+	786.6007	4.79
**PC 36:3**	[M+H]+	784.5851	4.39
**PC 36:4**	[M+H]+	782.5694	4.22
**PC 36:5**	[M+H]+	780.5538	4.16
**PC 38:2**	[M+H]+	814.632	5.48
**PC 38:3**	[M+H]+	812.6164	5.09
**PC 38:4**	[M+H]+	810.6007	4.87
**PC 38:5**	[M+H]+	808.5851	4.36
**PC 38:6**	[M+H]+	806.5694	4.22
**PC 40:4**	[M+H]+	838.632	5.45
**PC 40:5**	[M+H]+	836.6164	5.03
**PC 40:6**	[M+H]+	834.6007	4.87
**SM 32:0**	[M+H]+	677.5592	3.46
**SM 32:1**	[M+H]+	675.5436	3.31
**SM 32:2**	[M+H]+	673.5279	3.10
**SM 33:1**	[M+H]+	689.5592	3.50
**SM 34:1**	[M+H]+	703.5749	3.70
**SM 34:2**	[M+H]+	701.5592	3.44
**SM 35:1**	[M+H]+	717.5905	3.94
**SM 36:0**	[M+H]+	733.6218	4.42
**SM 36:1**	[M+H]+	731.6062	4.19
**SM 36:2**	[M+H]+	729.5905	3.87
**SM 38:1**	[M+H]+	759.6375	4.85
**SM 38:2**	[M+H]+	757.6218	4.43
**SM 39:1**	[M+H]+	773.6531	5.28
**SM 40:1**	[M+H]+	787.6688	5.70
**SM 40:2**	[M+H]+	785.6531	5.17
**SM 41:1**	[M+H]+	801.6844	6.11
**SM 41:2**	[M+H]+	799.6688	5.62
**SM 42:1**	[M+H]+	815.7001	6.29
**SM 42:2**	[M+H]+	813.6844	5.79
**SM 42:3**	[M+H]+	811.6688	5.24

**SM 43:1**	[M+H]+	829.7157	6.38
**SM 43:2**	[M+H]+	827.7001	6.04
**TG 46:0**	[M+NH4]+	796.7389	8.40
**TG 46:1**	[M+NH4]+	794.7232	8.13
**TG 46:2**	[M+NH4]+	792.7076	7.89
**TG 47:0**	[M+NH4]+	810.7545	9.17
**TG 47:1**	[M+NH4]+	808.7389	8.81
**TG 48:0**	[M+NH4]+	824.7702	8.81
**TG 48:1**	[M+NH4]+	822.7545	8.50
**TG 48:2**	[M+NH4]+	820.7389	8.22
**TG 48:3**	[M+NH4]+	818.7232	7.99
**TG 50:0**	[M+NH4]+	852.8015	9.29
**TG 50:1**	[M+NH4]+	850.7858	8.91
**TG 50:2**	[M+NH4]+	848.7702	8.60
**TG 50:3**	[M+NH4]+	846.7545	8.33
**TG 50:4**	[M+NH4]+	844.7389	8.09
**TG 51:2**	[M+NH4]+	862.7858	8.80
**TG 51:3**	[M+NH4]+	860.7702	8.69
**TG 52:1**	[M+NH4]+	878.8171	9.41
**TG 52:2**	[M+NH4]+	876.8015	9.01
**TG 52:3**	[M+NH4]+	874.7858	8.72
**TG 52:4**	[M+NH4]+	872.7702	8.47
**TG 52:5**	[M+NH4]+	870.7545	8.24
**TG 54:2**	[M+NH4]+	904.8328	9.53
**TG 54:3**	[M+NH4]+	902.8171	9.13
**TG 54:4**	[M+NH4]+	900.8015	8.83
**TG 54:5**	[M+NH4]+	898.7858	8.56
**TG 54:6**	[M+NH4]+	896.7702	8.31
**TG 54:7**	[M+NH4]+	894.7545	8.10
**TG 56:6**	[M+NH4]+	924.8015	9.12
**TG 56:7**	[M+NH4]+	922.7858	8.74
**TG 58:8**	[M+NH4]+	948.8015	9.00

Table S3. Relative concentrations of the 45 lipid species increased in the LTEC-losing group when compared to the LTEC-extreme individuals.

Lipid compunds	LTEC-losing	SEM	LTEC-extreme	SEM	Lipid compunds	LTEC-losing	SEM	LTEC-extreme	SEM
**SM (32:1)**	0,2116	0,0153	0,1373	0,0161	**PC (35:1)**	0,0115	0,0011	0,0074	0,0011
**SM (34:2)**	0,3626	0,0294	0,2671	0,0299	**PC (40:6)**	0,1198	0,0130	0,0694	0,0125
**SM (32:0)**	0,0051	0,0004	0,0035	0,0004	**PC (38:4)**	0,4598	0,0357	0,3157	0,0355
**SM (33:1)**	0,0985	0,0062	0,0730	0,0063	**SM (38:1)**	0,2804	0,0219	0,2095	0,0222
**PC (32:2)**	0,0129	0,0025	0,0067	0,0025	**PC (40:5)**	0,0204	0,0024	0,0109	0,0023
**PC (34:4)**	0,0039	0,0005	0,0020	0,0005	**PC (38:3)**	0,1795	0,0138	0,1281	0,0135
**PC (30:0)**	0,0132	0,0038	0,0057	0,0038	**PC (38:2)**	0,0182	0,0014	0,0123	0,0014
**PC (34:3)**	0,0447	0,0087	0,0251	0,0087	**SM (42:2)**	1,8332	0,1065	1,4279	0,1067
**PC (33:2)**	0,0092	0,0010	0,0054	0,0010	**TG (46:2)**	0,0646	0,0315	0,0312	0,0315
**SM (35:1)**	0,0600	0,0042	0,0420	0,0043	**TG (48:3)**	0,0866	0,0448	0,0340	0,0448
**PC (36:5)**	0,0982	0,0160	0,0309	0,0135	**TG (54:7)**	0,0721	0,0305	0,0244	0,0305
**PC (35:4)**	0,0031	0,0004	0,0016	0,0004	**CE (20:5)**	0,4340	0,0814	0,1214	0,0767
**PC (31:0)**	0,0022	0,0003	0,0012	0,0003	**CE (18:3)**	0,3544	0,0347	0,2310	0,0289
**PC (33:1)**	0,0078	0,0011	0,0044	0,0011	**CE (22:6)**	0,3444	0,0438	0,1833	0,0423
**SM (36:1)**	0,4317	0,0284	0,3281	0,0282	**TG (54:6)**	0,2590	0,0904	0,1054	0,0905
**PC (36:4)**	0,8663	0,0650	0,5788	0,0680	**CE (20:4)**	3,1239	0,2282	2,2060	0,2279
**PC (32:0)**	0,0699	0,0099	0,0443	0,0101	**TG (56:7)**	0,2041	0,0614	0,0843	0,0617
**PC (36:3)**	0,8228	0,0694	0,5940	0,0704	**CE (20:3)**	0,2072	0,0184	0,1447	0,0181
**SM (36:0)**	0,0196	0,0024	0,0126	0,0024	**TG (56:6)**	0,2103	0,0628	0,1014	0,0631
**PC (35:2)**	0,0291	0,0026	0,0206	0,0027	**CE (16:0)**	0,2344	0,0134	0,1741	0,0133
**SM (38:2)**	0,0938	0,0083	0,0705	0,0084	**TG (58:8)**	0,0483	0,0191	0,0164	0,0192
**PC (33:0)**	0,0023	0,0002	0,0015	0,0002	**CE (18:1)**	1,3846	0,1129	1,0141	0,1112
**PC (38:5)**	0,0559	0,0074	0,0252	0,0056					

Supplementary Figure 1. Overlapped extracted ion chromatograms (EIC) of metabolites analyzed by GC-EI-QTOF-MS.

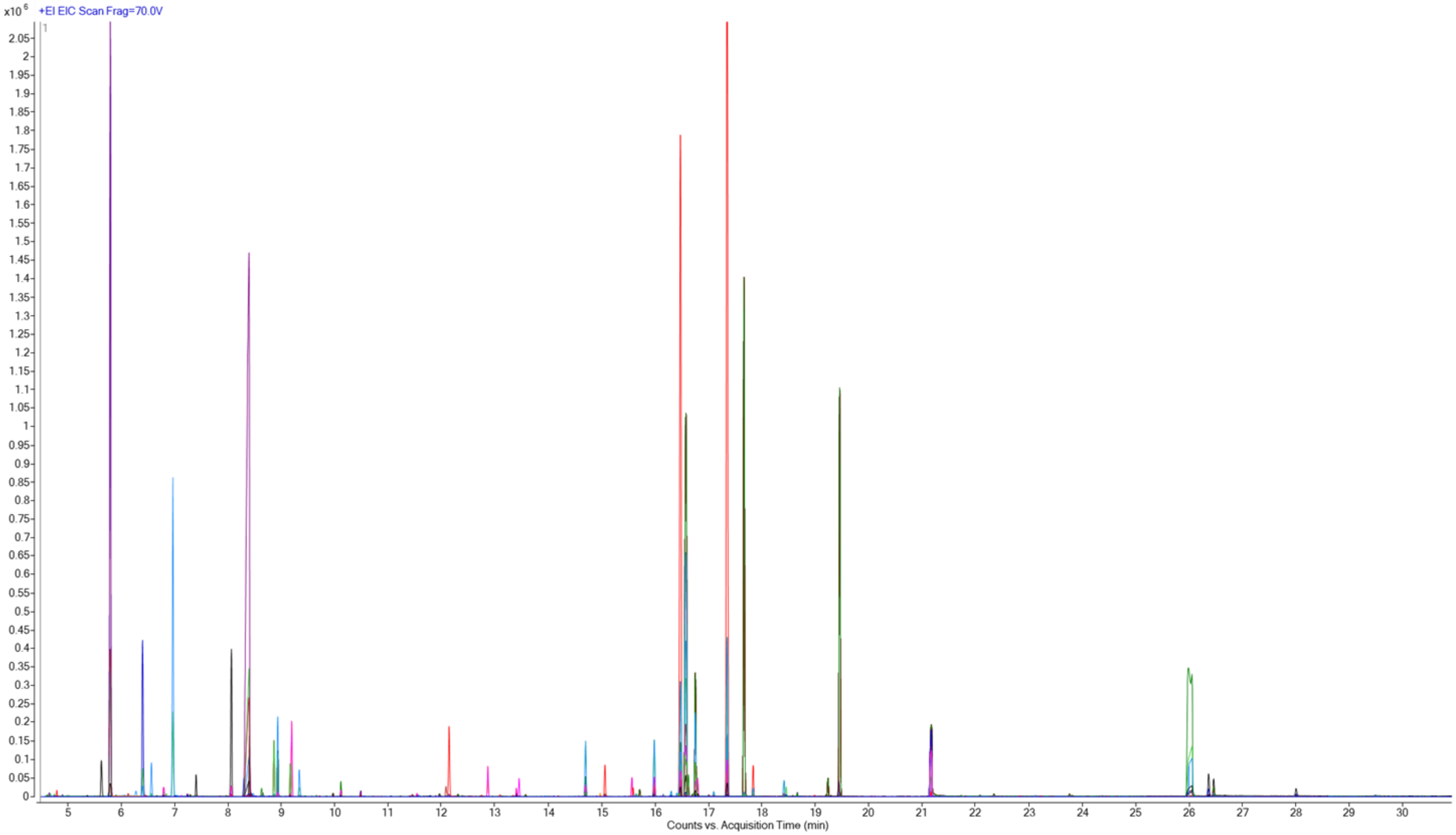

Supplementary Figure 2. Overlapped extracted ion chromatograms (EIC) of lipids analyzed by LC-ESI-QTOF-MS using the accurate mass (m/z) from pseudomolecular ions. LCP, lysophosphatidylcholines; PC, phosphatidylcholines; SM, sphingomyelins; DG, diglyceride; ChoE/CE, cholesteroyl esthers; TG, triglycerides.

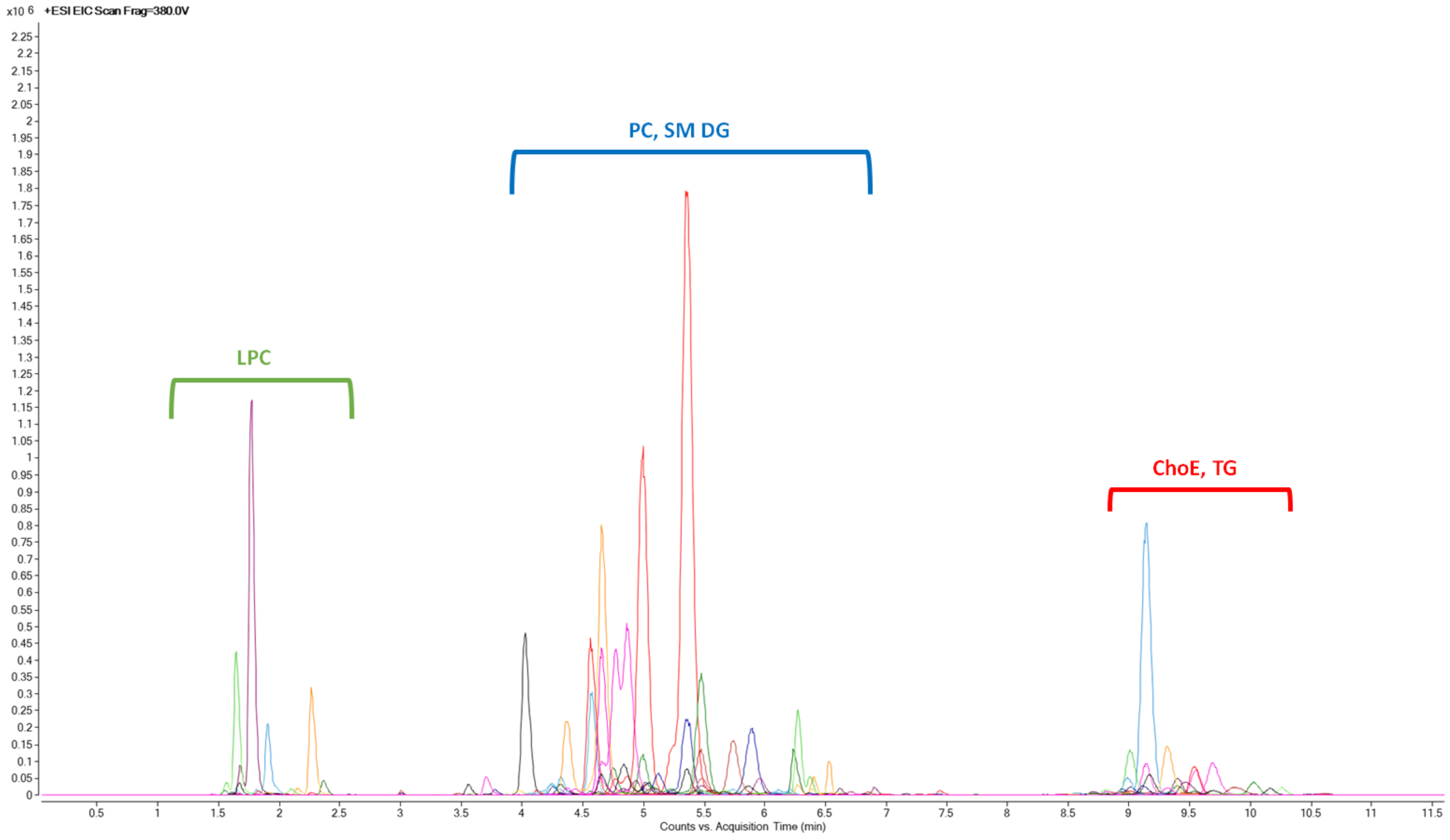

2 ECRIS integrated in the Spanish AIDS Research Network

a. Clinical Centres and Research Groups contributing to ECRIS

Hospital Universitario de Valme (Sevilla): Juan Antonio Pineda, Pilar Rincón, Juan Macías Sanchez, Luis Miguel Real, Anais Corma Gomez, Marta Fernandez Fuertes, Alejandro Gonzalez-Serna.

Hospital General Universitario Santa Lucía (Cartagena): Onofre Juan Martínez, Lorena Martinez, Francisco Jesús Vera, Josefina García, Begoña Alcaraz, Amaya Jimeno.

Hospital Clinic de Barcelona (Barcelona): José M. Miró, Christian Manzardo, Laura Zamora, Iñaki Pérez, Mª Teresa García, Carmen Ligero, José Luis Blanco, Felipe García-Alcaide, Esteban Martínez, Josep Mallolas, José M. Gatell.

Hospital General Universitario de Alicante (Alicante): Joaquín Portilla, Irene Portilla, Esperanza Merino, Gema García, Iván Agea, José Sánchez-Payá, Juan Carlos Rodríguez, Lina Gimeno, Livia Giner, Melissa Carreres, Sergio Reus, Vicente Boix, Diego Torrus, Verónica Pérez Esquerdo, Julia Portilla Tamarit.

Hospital Universitari de Bellvitge (Hospitalet de Llobregat): Daniel Podzamczer, Arkaitz Imaz, Juan Tiraboschi, Ana Silva, María Saumoy, Paula Prieto, Sofía Scevola.

Hospital Universitario de Canarias (Santa Cruz de Tenerife): Juan Luís Gómez Sirvent, Jehovana Hernández, Ana López Lirola, Dácil García, Felicitas Díaz-Flores, María del Mar Alonso, Ricardo Pelazas, María Remedios Alemán.

Hospital Carlos III (Madrid): Vicente Soriano, Pablo Labarga, Pablo Barreiro, Pablo Rivas, Francisco Blanco, Luz Martín Carbonero, Eugenia Vispo, Carmen Solera.

Hospital Universitario Central de Asturias (Oviedo): Victor Asensi, María Eugenia Rivas Carmenado, Tomas Suarez-Zarracina Secades.

Hospital Doce de Octubre (Madrid): Federico Pulido, Rafael Rubio, Otilia Bisbal, Mª Asunción Hernando, David Rial Crestelo, María de Lagarde, Rebeca Font, Octavio Arce, Adriana Pinto, Laura Bermejo, Mireia Santacreu.

Hospital Universitario Donostia (San Sebastián): Jose Antonio Iribarren, MªJosé Aramburu, Julio Arrizabalaga, Xabier Camino, Francisco Rodríguez-Arrondo, Miguel Ángel von Wichmann, Lidia Pascual Tomé, Miguel Ángel Goenaga, Mª Jesús Bustinduy, Harkaitz Azkune, Maialen Ibarguren, Xabier Kortajarena, Mª Pilar Carmona Oyaga, Ainhoa Garaialde Fernandez.

Hospital General Universitario de Elche (Elche): Félix Gutiérrez, Catalina Robledano, Mar Masiá, Sergio Padilla, Araceli Adsuar, Rafael Pascual, Marta Fernández, Antonio Galiana, José Alberto García, Xavier Barber, Vanessa Agullo Re, Javier Garcia Abellan, Reyes Pascual Pérez, Guillermo Telenti , Lucia Guillén, Angela Botella.

Hospital Germans Trías i Pujol (Badalona): Roberto Muga, Arantza Sanvisens, Daniel Fuster.

Hospital General Universitario Gregorio Marañón (Madrid): Juan Berenguer, Isabel Gutierrez Cuellar, Juan Carlos López Bernaldo de Quirós, Margarita Ramírez, Belén Padilla, Paloma Gijón, Teresa Aldamiz-Echevarría, Francisco Tejerina, Cristina Diez, Leire Pérez Latorre, Chiara Fanciulli, Saray Corral Carretero.

Hospital Universitari de Tarragona Joan XXIII, IISPV, Universitat Rovira i Virgili (Tarragona): Francesc Vidal, Anna Marti, Joaquín Peraire, Consuelo Viladés, Sergio Veloso, Montserrat Vargas, Montserrat Olona,

Anna Rull, Verónica Alba, Miguel López-Dupla, Elena Yeregui, Jenifer Masip, Graciano García-Pardo, Frederic Gomez-Bertomeu.

Hospital Universitario La Fe (Valencia): Marta Montero Alonso, Sandra Cuéllar Tovar, Marino Blanes Juliá, María Tasias Pitarch, Eva Calabuig Muñoz , Miguel Salavert Lletí , Juan Fernández Navarro.

Hospital Universitario La Paz/IdiPaz (Madrid): Juan González-Garcia, Ana Delgado Hierro, Francisco Arnalich, José Ramón Arribas, Jose Ignacio Bernardino de la Serna, Juan Miguel Castro, Luis Escosa, Pedro Herranz, Victor Hontañón, Silvia García-Bujalance, Milagros García López-Hortelano, Alicia González-Baeza, Maria Luz Martín-Carbonero, Mario Mayoral, Maria Jose Mellado, Rafael Esteban Micán, Rocio Montejano, María Luisa Montes, Victoria Moreno, Ignacio Pérez-Valero, Berta Rodés, Guadalupe Rúa Cebrián, Talia Sainz, Elena Sendagorta, Eulalia Valencia, Carmen Busca, Joanna Cano, Julen Cardiñanos, Rosa de Miguel.

Hospital de la Princesa (Madrid): Ignacio de los Santos Gil, Alejandro de los Santos San Frutos, Jesús Sanz Sanz, Lucio Garcia-Fraile Fraile, Enrique Martín Gayo, Ildefonso Sánchez-Cerrillo, Marta Calvet i Mirabent.

Hospital San Pedro-CIBIR (Logroño): José Antonio Oteo, José Ramón Blanco, Valvanera Ibarra, Luis Metola, Mercedes Sanz, Laura Pérez-Martínez.

Complejo Hospitalario de Navarra (Pamplona): María Rivero, Beatriz Piérola Ruiz de Galarreta, Maider Goikoetxea Peñagarikano, María Gracia Ruiz de Alda, Carlos Ibero Esparza, Estela Moreno García, Jesús Repáraz.

Hospital Parc Taulí (Sabadell): Gemma Navarro, María José Amengual, Manel Cervantes Garcia, Sonia Calzado Isbert, Marta Navarro Vilasaro, Belen Lopez Garcia.

Hospital Ramón y Cajal (Madrid): Santiago Moreno, Santos del Campo, José Luis Casado Osorio, Fernando Dronda Nuñez, Ana Moreno Zamora, Maria Jesús Pérez Elías, Carolina Gutiérrez, Nadia Madrid, Sergio Serrano Villar, Maria Jesús Vivancos Gallego, Javier Martínez Sanz, Tamara Velasco, Alejandro Vallejo, Matilde Sanchez Conde, Jose Antonio Pérez Molina.

Hospital Reina Sofía (Murcia): Enrique Bernal, Antonia Alcaraz, Joaquín Bravo Urbieta, Angeles Muñoz Perez, Cristina Tomás Jimenez, Monica Martinez Martinez, Maria Jose Alcaraz, Maria del Carmen Villalba.

Hospital San Cecilio (Granada): Federico García, Clara Martínez, José Hernández Quero, Leopoldo Muñoz Medina, Marta Alvarez, Natalia Chueca, David Vinuesa García, Adolfo de Salazar Gonzalerz, Ana Fuentes Lopez.

Centro Sanitario Sandoval (Madrid): Jorge Del Romero Guerrero, Montserrat Raposo Utrilla, Carmen Rodríguez, Teresa Puerta, Juan Carlos Carrió, Mar Vera, Juan Ballesteros, Oskar Ayerdi.

Hospital Son Espases (Palma de Mallorca): Melchor Riera, María Peñaranda, Mª Angels Ribas, Antonia Campins, Carmen Vidal, Francisco Fanjul, Javier Murillas, Francisco Homar, Helem H Vilchez, Maria Luisa Martin, Antoni Payeras.

Hospital Universitario Virgen del Rocío (Sevilla): Luis Fernando López-Cortés, Silvia Llaves, Nuria Espinosa.

b. Research groups:

Hospital General Universitario Gregorio Marañón e Insituto de Investigación Sanitaria Gregorio Marañón. Maria Angeles Muñoz-Fernández, Laura Tarancon-Diez, Jose Luis Jimenez, Daniel Sepúlveda, Rafael Ceña, Isabel García Merino, Irene Consuegra.

Hospital Clinic. Agathe León, Montse Plana, Nuria Climent, Felipe García.

Hospital Joan XXIII. Francesc Vidal, Anna Marti, Joaquín Peraire, Consuelo Viladés, Sergio Veloso, Montserrat Vargas, Montserrat Olona, Anna Rull, Verónica Alba, Miguel López-Dupla, Elena Yeregui, Jenifer Masip, Graciano García-Pardo, Frederic Gomez-Bertomeu.

IIS-Fundacion Jimenez Díaz, UAM. Jose Miguel Benito, Norma Rallón, Clara Restrepo, Marcial García, Alfonso Cabello, Miguel Gorgolas.

Centro Sandoval. Jorge Del Romero, Carmen Rodríguez, Mar Vera.

Fundacion IRSI CAIXA. José Esté, Esther Ballana, Miguel Angel Martinez, S Franco, María Nevot, Julia G. Prado, Esther Jiménez

Hospital Ramón y Cajal. Alejandro Vallejo, Beatriz Sara Sastre, Santiago Moreno.

Virologia Molecular ISCIII. Maria Pernas, Concepción Casado, Cecilio López Galíndez

Infeccion viral e Inmunidad. ISCIII. Salvador Resino

Inmunopatología del SIDA. ISCIII. Laura Capa, Mayte Perez-Olmeda, Pepe Alcami

Mutacion y evolución de virus. Univ Valencia. Rafael Sanjuán, José Manuel Cuevas

Hospital Universitario Doce de Octubre (Madrid): Rafael Rubio, Federico Pulido, Otilia Bisbal, Mª Asunción Hernando, Mariano Matarranz, María Lagarde, Lourdes Domínguez.

Universidad de la Laguna. Agustín Valenzuela-Fernández.

Hospital Virgen del Rocio: Ezequiel Ruiz-Mateos, María Reyes Jiménez-León, Carmen Gasca-Capote, Alberto Pérez-Gómez, Mohamed Rafii-El-Idrissi Benhnia, Alicia Gutierrez-Valencia, María Trujillo, Ana Serna-Gallego, Esperanza Muñoz-Muela, Silvia Llaves, Cristina Roca-Oporto, Nuria Espinosa, Luis Fernando López-Cortés.
